# How economic implications of gender gaps in employment affect global health equity

**DOI:** 10.2471/BLT.23.291271

**Published:** 2024-02-01

**Authors:** Michelle McIsaac, Felicia Marie Knaul

**Affiliations:** a Health Workforce Department, World Health Organization, Avenue Appia 20, 1211 Geneva 27, Switzerland.; b Institute for Advanced Study of the Americas, University of Miami, Miami, United States of America.

Professor Claudia Goldin won the 2023 Sveriges Riksbank prize in economic sciences in memory of Alfred Nobel for her pioneering research on gender gaps in the labour market, uncovering how gender influences the interface between work and how it is valued.^1^ The history of the Nobel prize epitomizes the evolution and persistence of the gender earnings gap and obstacles women face in the labour market. Since 1969, there have been 93 economics laureates; Goldin is the third woman and the first female solo awardee.^2^ The health and care sector exemplifies the gender gaps in the labour market that Goldin discusses in her work.

While this theme issue of the *Bulletin of the World Health Organization* looks to uncover health inequities leading to unfair and avoidable differences in health outcomes, Goldin’s work suggests that a foundational inequity could be at the root of all health inequities: the low value placed on those who deliver health, and their work. Gendered jobs and the devaluation of feminized work are common across the health and care sector, and the relationship between this sector and the gender earnings gap is more nuanced and complex than for many other sectors.^3^

Structural shifts have taken place in the way health is delivered that have led to some improvements in gender equality for health workers. An example Goldin cites is the reduction of self-employed pharmacists in the United States of America that led to pharmacy becoming more gender equal.^4^ However, the often-avoided issue that a large amount of health and care work is undervalued and very often unpaid remains.^5^ From a health systems perspective, low and unpaid work is often considered a cost-effective mode of delivering health, but this undervaluation of care work is at the root of the problem of balancing career and family.^4^ Unpaid care work, as well as many jobs undertaken in the home that promote health and prevent disease such as cooking and cleaning, are gendered in terms of earnings, hours, tasks and occupations. Jobs in the health and care sector often require high levels of education, skill and commitment; however, they also remain undervalued in terms of compensation compared to jobs with similar labour market profiles in other sectors.^3^ This divide impacts the health and care sector and the entire economy. 

Care work, and who performs it, is a root cause of gender inequality in the labour market.^6^ The gendered nature of health and care jobs is well documented, with two thirds of paid workers in the health and care sector across the world being women.^3^^,^^5^^–^^7^ However, Goldin’s historical approach to analysing the evolution of the gender earnings gap provides critical insights that demand a better understanding of this sector. Women in almost all health sector contexts still face many barriers in securing wages and working conditions that equal those of their male counterparts, a reality hidden behind encouraging trends in global averages – for example the increases in women studying and working in medicine, a historically high-paying, high-status, male-dominated occupation. Furthermore, health sector leadership and hence decision-making continues to be dominated by men. Global averages also mask regional inequalities and contexts that prevent women from accessing paid employment. For example, men are the majority of health and care workers in the World Health Organization (WHO) Eastern Mediterranean Region, and across most low-income countries.^3^^,^^6^ As well, women’s access to quality health care is an important determinant of educational outcomes and labour market productivity.

The WHO council on the economics of health for all is calling on governments to rethink value, and reshape and redirect the economy based on social and planetary well-being using new metrics.^8^ Yet, the devaluation of work in the care economy and of women who work in the health and care sector remains a deeply ingrained and persistent obstacle to societal well-being as well as to greater equity and efficiency.^9^

Goldin’s work underscores the necessary examination of the perception and remuneration of work within the health and care sectors. The needed re-evaluation goes beyond acknowledging that gender plays a role in how we value health and care work; it demands actionable change to ensure that the health and care sector makes gender inequality costly to sustain. The status quo, that is, the undervaluation of women and of health and care work, negatively affects women’s empowerment, economic growth and development, and population health. The health and care sector, and hence the production of population health globally, must be transformed from being a sustainer of gender inequality into a catalyst and driver of gender equality. Health systems need to recognize, value and invest in health and care work to deliver on the promise of gender equality and universal health coverage.
